# Comparison between the synthesis of gold nanoparticles with sodium citrate and sodium tetraboreto

**DOI:** 10.1186/1753-6561-8-S4-P252

**Published:** 2014-10-01

**Authors:** Adilson R Prado, Jairo P Oliveira, Wanderson J Keijok, Bárbara Altoé Milaneze, Breno V Nogueira, Marco CC Guimarães, Maria J Pontes, Moises RN Ribeiro

**Affiliations:** 1 Telecommunications Laboratory (LabTel), DEL/CT, Federal University of Espírito Santo (UFES), Vitória, ES, Brazil; 2 Federal Institute of the Espírito Santo (IFES), Vitória, ES, Brazil; 3 Laboratory Cellular Ultrastructure (CCS), Federal University of Espírito Santo (UFES), Vitória, ES, Brazil

## Background

With gold nanoparticles (AuNPs) is possible to develop nanoscale devices that can interact with chemical and biological systems. The phenomenon explored in these nanosystems is called Localized Surface Plasmon Resonance (LSPR), which promotes electromagnetic wave oscillation electronics on these small metallic structures. It is interesting to note that this resonance is directly linked to the size of the nanoparticles, the nature of the dielectric material and support environment where the device is being studied [1,2].

This work makes a comparison between results obtained in the synthesis of AuNP's reduction method using a Sodium Citrate (Na_3_C_6_H_5_O_7_) and Sodium Borohydride (NaBH_4_). Was expected to demonstrate the viability of these two reducing agents and highlight the potential differences obtained in each of the mechanisms. Since knowledge is the ability of stabilizing citrate ions and the strong reducing action of NaBH_4 _[3]. The dominance of this knowledge will provide the development of systems with particle size specific for various applications in biosensors.

## Methods

In these experiments consisted initially in the preparation of the following aqueous solutions: 100 mL Tetrachloroauric Acid (HAuCl_4_) 2.5 × 10^-4 ^M, 5 mL of 1% NaBH_4 _and 5 mL of Na_3_C_6_H_5_O_7 _also 1%.

The synthesis of nanoparticles were carried out simultaneously with the reducing agents, for it was placing 50.0 mL of HAuCl4 solution in two Erlenmeyer and the following adding agent reducing in agitation. The system with sodium citrate was put into a heating temperature of 65 ° C with NaBH_4 _has not been heated. The reaction time for both was 25 min, but the use of heating in half with Na_3_C_6_H_5_O_7 _was off to achieve 10 min of reaction. The procedure used here was based on literature data, with some adaptations [3].

The samples produced AuNP's had their optical properties assessed by UV-Vis spectrophotometry. The size and morphology of AuNP's were examined by Transmission Electron Microscopy (TEM) (JEM-1400, JEOL/USA Inc.).

## Results and conclusion

The UV-Vis spectrophotometry shows the absorption of the samples obtained in experiments, it is noted that the easy synthesis with NaBH_4 _did not generate nanoparticles with a reasonable size for the LSPR occur. Phenomenon responsible for the peak at 530 nm in the spectrum of sodium citrate (Na_3_C_6_H_5_O_7_).

Due to capacity reduction of NaBH_4 _lot nuclei initiators nanoparticles were generated, but reduced in size. Na_3_C_6_H_5_O_7 _already has a good ability to stabilize, thus required temperature rise in the synthesis process to increase capacity reduction. Nuclei being formed ions favor the stabilization phase of growth, thus generating nanostructures favorable process RPSL to 530 nm.
